# Effects of a Short Mobile Intervention on Digital Health Literacy in Adolescents and Teachers: Randomized Controlled Trial

**DOI:** 10.2196/96284

**Published:** 2026-07-21

**Authors:** Rebekka Schröder, Tim Hamer, Victoria Kruzewitz, Ralf Suhr, Lars König

**Affiliations:** 1 Stiftung Gesundheitswissen Berlin Germany

**Keywords:** digital health literacy, adolescents, health literacy, online intervention, e-learning, randomized controlled trial, schools, health promotion, short mobile intervention

## Abstract

**Background:**

Digital health literacy is an essential skill for processing health-related information in today’s technology-driven society. Recent literature highlights deficits in digital health literacy among adolescents and legislative initiatives to secure its promotion have been introduced (eg, in the German Social Code Book V). However, few interventions target adolescents; existing programs often overlook critical aspects like graph literacy or lack applicability in schools and rigorous scientific evaluation. To overcome these limitations, short mobile interventions—optimized for mobile devices and flexible deployment in schools—were developed to promote digital health literacy.

**Objective:**

This study aimed to evaluate the effectiveness of one of these interventions (focusing on graph-based health information) and to assess its quality and applicability within educational settings as a proof-of-concept for the further development of similar interventions for adolescents.

**Methods:**

A randomized-controlled pre-post trial with an active control group was conducted across two independent samples in a web-based survey: adolescents in secondary schools (14-17 years) and teachers. Participants in each sample were randomly assigned (1:1 allocation) to the experimental or control group. Following baseline data collection (T1), participants completed their assigned interventions, immediately followed by postassessments (T2). The experimental intervention is a mobile-friendly e-learning module (approximately 10 minutes) featuring interactive tasks focused on interpreting graph-based health information. The control intervention followed the same design and logic but covered a different topic. The primary outcome was subjective knowledge regarding the intervention’s topics. Secondary outcomes included objective knowledge (quiz scores) and 2 adapted subscales of the Digital Health Literacy Instrument (DHLI: “evaluating reliability” and “determining relevance”). Educational quality, visual aesthetics, and practical applicability were also assessed at T2.

**Results:**

Among adolescents (experimental group: n=251; control group: n=249), a significant interaction effect was observed only for objective knowledge (*F*_1,498_=70.42, η²_p_=.124; *P*<.001), but not for the other three outcomes (subjective knowledge: *F*_1,498_=2.04, η²_p_=.004; *P*=.15; evaluating reliability: *F*_1,498_=0.28, η²_p_<.001; *P*=.60; determining relevance: *F*_1,498_=0.25, η²_p_<.001; *P*=.62), meaning that the intervention improved fact-based but not subjective knowledge and no broader health literacy related skills. For teachers (experimental group: n=301; control group: n=302), the results showed significant interaction effects for subjective knowledge (*F*_1,601_=32.42, η²_p_=.051; *P*<.001), objective knowledge (*F*_1,601_=128.79, η²_p_=.177; *P*<.001), and one facet of digital health literacy (evaluating reliability: *F*_1,601_=6.21, η²_p_=.010; *P*=.01), while the interaction effect for “determining relevance” was not significant (F_1,601_=1.97, η²_p_=.003; *P*=.16), suggesting that the intervention improved teachers’ knowledge and one facet of digital health literacy. Both samples evaluated the visual and educational quality of the intervention as positive.

**Conclusions:**

This study demonstrates that short mobile interventions hold significant promise for the promotion of digital health literacy (eg, graph literacy) within school environments. These interventions can effectively teach objective knowledge to adolescents and teachers, but improving their digital health literacy may require more comprehensive or multimodule approaches.

**Trial Registration:**

German Clinical Trials Register DRKS00037830; https://drks.de/search/de/trial/DRKS00037830/details

## Introduction

Health literacy is a fundamental determinant of health—both at an individual and population level—as it enables individuals to access, understand, appraise, and apply health information to make informed decisions for their health and well-being [1]. Strengthening health literacy is therefore widely recognized as a key strategy to promote long-term public health and reduce costs in the health care system [1-4].

In light of the ongoing and rapidly growing digitization, digital health literacy (or eHealth literacy) is acknowledged as a critical element to contribute to overall well-being and health, which has led to its characterization as a “super determinant of health” [5,6]. While initially defined as the ability to find, understand, appraise, and apply electronic health information [6], recent definitions expand this to a multifactorial skillset. This modern view emphasizes transactional and contextual aspects, shifting the focus toward the user’s ability to actively exchange information and interact with others to manage health effectively [7,8]. Higher digital health literacy has been associated with more successful use of digital health resources, adoption of more health-promoting behaviors, better individual health management and participation in medical decisions, and a higher quality of life [9,10]. Importantly, digital health literacy is not a singular skill but the integration of different literacies, including general health literacy, media literacy, data literacy, and scientific literacy [6,9]. However, despite its importance, levels of digital health literacy remain insufficient in many segments of society [9-11]. For example, only about 65% of children and adolescents from Germany have sufficient levels of digital health literacy [11]. At the same time, this deficit is typically not adequately dealt with in schools [12]. To address this gap—and in line with World Health Organization recommendations to strengthen both general and digital health literacy [13,14]—national authorities in Germany have introduced legislation in the Social Code Book V to legally require statutory health insurance companies in Germany to develop and implement programs that promote digital health literacy [15,16]. This has led to first interventions being created, evaluated, and launched [17-19].

However, previous efforts to enhance digital health literacy are often quite extensive, which may hinder their practical implementation in classroom settings or integration into existing educational curricula [20]. For example, one comprehensive health literacy program for vocational schools entails a project day in addition to an 8-week online course [21], while others encompass comprehensive e-learning sessions of up to 90 minutes [17-19]. Moreover, many existing interventions have primarily focused on the promotion of the processing of health-related information in textual or verbal form [17-19]. Adopting this narrow focus has led to the relative neglect of other integral aspects of digital health literacy, namely scientific, media, basic, and information literacy [22,23]. These competencies include the understanding and processing of information presented in nontextual formats, such as numerical data, tables, and graphs [24,25], which is tightly connected to the construct of data literacy, a critical life skill highly relevant for health-related decisions and beyond [26]. Hence, a broader and more integrated promotion of digital health literacy is needed that considers all of its integral elements. Therefore, the present study places a particular focus on graph-based health information. Beyond content, greater emphasis should also be placed on new formats and approaches related to the implementation of interventions, for example on the media and resources used to promote digital health literacy. These may include analog materials and digital learning platforms [27]. In recent years, the use of digital technologies with interactive elements has emerged as a promising approach, and it was recommended to consider different technological methods in the development of new interventions, including the integration of gamification elements and the use of mobile platforms [27], which may lead to better engagement and learning outcomes. Following these practical recommendations and fulfilling public policy recommendations, the nonprofit foundation *Stiftung Gesundheitswissen* has recently developed several short mobile interventions as tools to promote digital health literacy. Each intervention focuses on a specific health-related topic, for example, the promotion of graph literacy or the understanding of average values, and incorporates gamification elements [27-30]. The short mobile interventions were developed so that they can be used in different school subjects and aligned with different curricula [20]. They are presented on mobile platforms that are both flexible and easily available, which might facilitate their implementation in schools [31]. Furthermore, they have the capacity to reach a significant number of users and can be updated easily.

As proof-of-concept for these newly developed short mobile interventions, the primary aim of the present study was to evaluate one of these interventions (ie, on understanding graph-based health information) concerning its effectiveness in promoting digital health literacy. Two target groups were selected: adolescents, aged 14-17 years, who represent the primary target group for the interventions, and teachers in German secondary schools who are experts on the subject and may act as facilitators for the implementation of the interventions in schools if they consider them suitable. By assessing both effectiveness and feasibility among these groups, this study contributes to the evidence base needed to inform future large-scale implementations of digital health literacy interventions in schools.

The following hypotheses were tested in a randomized-controlled pre-post study design in two independent samples of teachers and adolescents:

1. Completing the short mobile intervention improves self-reported knowledge on how to recognize good graphs in the participants in the experimental group compared to an active control group (hypothesis 1, primary outcome).
2. Completing the short mobile intervention improves objective knowledge on how to recognize good graphs in the participants in the experimental group compared to an active control group (hypothesis 2, secondary outcome).
3. Completing the short mobile intervention improves the digital health literacy of the participants in the experimental group compared to an active control group in the domains of “evaluating reliability” (hypothesis 3a, secondary outcome) and “determining relevance” (hypothesis 3b, secondary outcome).

Furthermore, to assess the practical feasibility of the intervention, secondary analyses explored how the participants evaluated the overall quality, applicability, visual aesthetics, and other core features after completion.

## Methods

### Ethical Considerations

The study was performed in accordance with the Declaration of Helsinki. A study protocol detailing objectives, procedures, materials, and measures was submitted to the Ethics Committee of the Berlin Medical Association prior to data collection. The ethics committee ruled that the nature of the project does not require professional ethical and legal consultation (reference number: Eth-KB-25-017, dated August 29, 2025). Following ethical review, the study was registered at the German Clinical Trials Register (DRKS00037830). Before participation, individuals were informed about the study’s aims, procedures, and data security measures. They were explicitly notified that participation was voluntary and that they could withdraw at any time without negative consequences. All participants provided informed consent via checkboxes on the survey platform; for participants aged 15 years and younger, additional informed consent was obtained from a parent or legal guardian. Privacy and confidentiality of the participants’ data and identity were maintained throughout the entire study procedure. Participants were compensated by the market research institute responsible for data collection.

### Target Populations, Recruitment, and Sample Size Considerations

This study was conducted in two independent samples of teachers and adolescents from Germany. Participants were recruited from a large online panel with approximately 150,000 participants that are—in turn—recruited exclusively offline via telephone. The panel is representative of the German-speaking population in Germany, aged 14 years and older. A range of quality assurance protocols ensure the prevention of duplicate panel participation. For the teachers’ sample, a random sample from all panelists working as teachers at secondary or vocational schools was drawn (ie, no primary school teachers). Before study onset, teacher and school status were verified by the participants via screening questions. Adolescents (aged 14-17 years) were recruited either directly from the panel via existing panel data or through their parents using a random selection process (specifically the “last birthday method”) to ensure representativeness. Parents were asked to let their children complete the questionnaire independently to maintain data integrity.

For the teachers’ sample, the following inclusion criteria were determined: informed consent, male, female or diverse gender, age 18 years and older, currently working as a teacher in a secondary school, sufficient knowledge of the German language, and living in Germany. For the adolescents’ sample, inclusion criteria were informed consent, male, female or diverse gender, age between 14 and 17 years, sufficient knowledge of the German language, and living in Germany. There were no exclusion criteria apart from not fulfilling the inclusion criteria.

Participants in both samples were invited via e-mail with a maximum of 2 reminders sent.

An a-priori sample size calculation was computed using G*Power (version 3.1.9.7) [32] with the following parameters for the interaction effect in an analysis of variance, which was the primary outcome in this study (see *Statistical Analysis*): α=.05, effect size η^2^_p_=.03 (*f*=0.176), power=.80. This resulted in a minimum sample size of 258 participants per sample. The small to medium effect size is based on previous studies of similar evaluations [17-19]. In order to ensure a sufficiently large number of cases even in the event of possible dropouts, we aimed to recruit approximately 500 individuals per sample. Data collection took place in October and November 2025.

### Intervention

The intervention evaluated here is a short interactive e-learning module, developed to enhance specific aspects of digital health literacy and to be used on mobile or larger devices. It takes approximately 10 minutes to complete. Short mobile interventions can be used as stand-alone interventions or to complement and foster topics already addressed in school lessons or other learning settings. This flexibility facilitates their implementation in blended-learning environments [33]. To enhance motivation and engagement of the users, the short mobile interventions incorporate elements of gamification [28-30], by using game design elements [34]. Specifically, the users win and lose points and get immediate feedback for their actions and decisions, and they are awarded a virtual trophy depending on their final score. These gamification elements can enhance motivation and engagement of the users to actively deal with the topics addressed in the modules [29,30]. The interventions combine interactive visual and written elements. They are complemented by optional sounds that can be deactivated and are not necessary for their successful completion, for example when used in larger classrooms. Several different short mobile interventions were recently developed, but only one was selected to be evaluated here in detail. This specific intervention focuses on how to recognize good graphs with the help of different criteria. Another intervention was presented to the control group that focuses on how to interpret average values in the context of health information (eg, arithmetic means). The interventions are structured in five parts. First, a short technical introduction and instructions are given to explain the structure and functions of the intervention, how to interact with it, and how the point system works. This technical introduction is followed by a topical introduction that is both target-group–oriented and problem-based. In the case of the short mobile intervention evaluated here, this topical introduction includes the presentation of two graphs that convey information about germs on different surfaces that the participants might encounter when scrolling through social media. After a brief introduction, the main part begins, in which they learn how to judge good graphs based on certain criteria, building the skillset to properly judge the two graphs from the introduction. This part consists of multiple exercises, has a progress bar, has points added and subtracted from a total score, and incorporates feedback elements to explain correct and incorrect responses. Five different learning objectives are pursued in this main part that are addressed in several different tasks, for example, multiple-choice questions, selection tasks, and slide bars:

1. Students learn important criteria for evaluating and recognizing good graphs by selecting them.
2. Students apply the criteria for recognizing good graphs by evaluating two graphs with regard to a specific criterion.
3. Students can recognize good graphs using different criteria by applying them to an example.
4. The students can recognize good graphs using different criteria by evaluating two graphs.
5. The students’ knowledge is consolidated by solving multiple-choice tasks on the topic.

**Figure 1:**
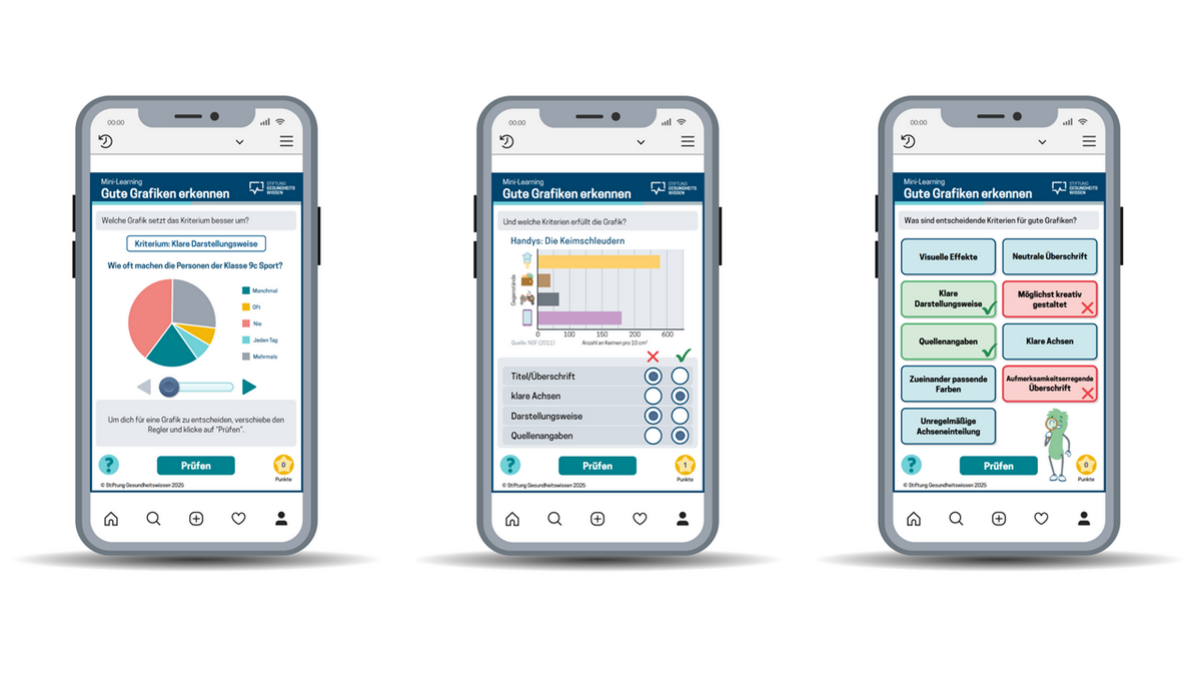
Screenshots of three tasks in the experimental short mobile intervention.

The final part includes brief feedback. Participants are awarded one of three trophies (bronze, silver, or gold) or no trophy according to their overall score. If they have not reached more than half of the total number of points, they do not receive a trophy and are invited to repeat the short mobile intervention to foster their knowledge. Screenshots of the intervention used in the experimental group can be found in Figure 1.

### Study Design and Procedure

This web-based study used a prospective randomized-controlled pre-post study design with one experimental group and an active control group. Group allocation was based on a computerized randomization procedure with an allocation ratio of 1:1. The randomization sequence was generated by the market research institute. Participants were blinded to the fact whether their respective mobile intervention was the experimental or the control intervention. At T1 (preassessment), all participants completed an online survey to obtain sociodemographic information, baseline levels of objective and subjective knowledge, and digital health literacy. Then, both groups conducted their respective short mobile intervention described above (see *Intervention*), which was directly integrated into the survey administration software. At T2 (postassessment), participants in both groups filled in the knowledge and digital health literacy questionnaires again. In addition, a number of questionnaires directly related to the intervention were assessed. These encompassed questions on quality, visual aesthetics, length, tempo, perceived difficulty, applicability in schools, implementation intentions, and motivation (for more details, refer to *Measures*).

### Measures

#### Sociodemographic Measures

At T1, basic sociodemographic information was collected in both samples and groups. Specifically, participants reported their gender, age, and the federal state and type of school they currently attend or teach at.

#### Knowledge

Knowledge on how to recognize good graphs was assessed on two levels: a subjective rating based on a self-report measure, and an objective measure using a knowledge quiz.

The self-report measure consisted of 12 items. Each item either starts with “I can explain…” or “I know…” and refers to the participants’ ability in evaluating graphs and recognizing good graphs, for example, knowing how to differentiate good and bad graphs or recognizing elements that constitute a good graph. Participants were asked to rate these items on a 6-point Likert scale (1 “strongly disagree,” 2 “disagree,” 3 “somewhat disagree,” 4 “somewhat agree,” 5 “agree,” and 6 “strongly agree”). A total score was computed as the mean value of these items with higher values denoting higher subjective knowledge. The self-reported knowledge was preregistered as the primary study outcome, while all other outcomes described below were secondary.

The objective knowledge quiz consisted of eight items covering the central aspects addressed in the short mobile intervention. Each item is a question with four response options. Participants were instructed to mark all correct responses. They were informed that one, two, three, or all four response options could be correct. They were given one point for each question that was answered correctly, that is, if all correct response options were selected and no false response option was selected. Points were added up across all items, resulting in a total score ranging from 0 to 8, with higher values denoting higher objective knowledge. In two of the items, participants were presented with graphs and were asked to select the correct statements concerning the interpretation of these graphs. The other items were more general and referred to the criteria for recognizing good graphs. All knowledge items can be found in Multimedia Appendix 1.

#### Digital Health Literacy

The “evaluating reliability” and “determining relevance” subscales of the Digital Health Literacy Instrument (DHLI) were adapted to assess aspects of digital health literacy [8,35]. The other subscales of the questionnaire were not used in the current study because they cover topics not addressed in the short mobile intervention evaluated here. The wording of all items was adjusted to refer to health-related graphs instead of health information in general. Each scale consists of three items that were rated on a four-point scale with the response options “very difficult” (1), “difficult” (2), “easy” (3), and “very easy” (4). A mean score was calculated for each subscale with higher values denoting higher digital health literacy in the respective field.

#### Postintervention Evaluation

Two validated questionnaires were presented to the participants at T2 to evaluate the user experience of the short mobile interventions. First, the German version of the “learning and value” subscale of the Student Evaluation of Educational Quality (SEEQ) instrument was used to assess the educational quality of the intervention [36,37]. This instrument was initially developed to assess students’ evaluation of teaching effectiveness in academic contexts. For the present study, the wording in the “learning and value” subscale was adapted to refer to the short mobile interventions. The questionnaire subscale entails five items that are rated on a scale ranging from “strongly disagree” (1) to “strongly agree” (5). A mean score was computed across these five items, with higher values representing higher quality.

In addition, the German version of the Visual Aesthetics of Websites Inventory (VisAWI) was used to assess the visual quality regarding the short mobile intervention [38]. The questionnaire has a total of 18 items that belong to four subscales: simplicity (5 items), diversity (5 items), colorfulness (4 items), and craftsmanship (4 items). The items are rated on a scale ranging from “strongly disagree” (1) to “strongly agree” (7). Eight items are reverse-coded and were re-coded before further analysis. A mean score for each subscale and an overall mean score across all items were computed. If the mean score exceeds 4.5, this can be interpreted as an overall positive evaluation of a website [39].

In addition, all participants were asked to rate the length, tempo, and difficulty of the short mobile interventions on three separate 3-point Likert scales (“too short,” “exactly right,” “too long”; “too fast,” “exactly right,” “too slow”; “too difficult,” “exactly right,” and “too easy”).

To assess the applicability of the short mobile interventions in school contexts, the adolescents and teachers were asked if they would complete the intervention in their free time, and if they would recommend them to friends or family members (three separate questions). In addition, only the adolescents were asked if they would feel motivated if these or similar short mobile interventions were used in (1) lessons, (2) as homework, (3) in project weeks, (4) in class chats or digital learning platforms, and (5) if their teachers would recommend them to them. Finally, only the teachers were asked if they would use these or similar short mobile interventions in (6) lessons, (7) as homework, (8) in project weeks, (9) in class chats or digital learning platforms, and if they would recommend them to (10) their colleagues or (11) their students. All these questions were answered on a 4-point Likert scale (“no,” “rather no,” “rather yes,” and “yes”).

### Statistical Analysis

Data were preprocessed using the statistical software SPSS (version 29.0.2.0; IBM Corp). All inferential analyses were then conducted with the statistical software JASP (version 0.19.1). Only participants with complete data (T1 and T2) were included in the analyses. To analyze the effectiveness of the intervention, mixed ANOVAs were computed with the between-subjects factor group (experimental group, control group) and the within-subjects factor time (T1, T2), separately for each dependent variable (total score subjective knowledge self-report, total score objective knowledge quiz, mean values for the DHLI “evaluating reliability” and “determining relevance” subscales) and each sample. If these resulted in significant interaction effects, additional 2-tailed *t* tests were computed for the direct comparison of each pair of factor levels. Effect sizes for the ANOVAs are reported as η^2^_p_. Effect sizes for the post hoc *t* tests are reported as Cohen *d*. For all analyses, *P* values are reported two-sided, and α=.05 was used as the significance threshold. *P* values were adjusted using a Bonferroni-correction for the post hoc *t* tests. Internal consistency reliability coefficients for the overall and subscale scores were computed as Cronbach α. These can be interpreted with the following rule of thumb: >.90 excellent, >.80 good, >.70 acceptable, >.60 questionable, >.50 poor and <.50 unacceptable [40].

## Results

### Sample Characteristics

In the adolescents’ sample, 500 individuals (n=260 female, n=232 male, n=8 with diverse gender) aged 14-17 years (mean 15.46, SD 1.11 years) participated in the study. The adolescents studied at different types of schools with the biggest proportion (n=313) attending *Gymnasium*, the secondary school to acquire a university entrance qualification.

In the teachers’ sample, 603 individuals (n=322 female, n=281 male) aged 20-69 years (mean 51.48, *SD* 9.11 years) participated in the study. The biggest proportions of teachers (n=170) taught at *Gymnasium* or at vocational schools (n=136).

**Figure 2:**
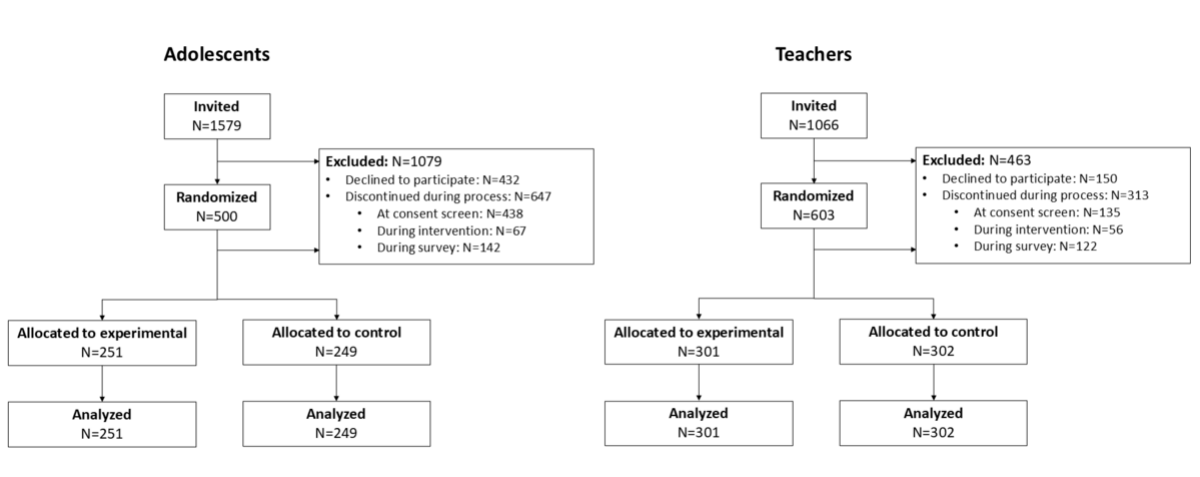
Flowchart across the study phases for the teachers’ and adolescents’ samples.

The number of participants across the study phases is presented in Figure 2. There were no significant differences regarding age (adolescents: *t*_498_=0.65, *d*=0.06; *P*=.52; teachers: *t*_601_=–0.65, *d*=–0.05; *P*=.52), nor gender distribution (adolescents: χ^2^_2_=0.65, *V*=0.04; *P*=.72; teachers: χ^2^_1_=0.002, *V*=0.002; *P*=.97) between the experimental and control groups in either sample.

### Quality of the Measures

Cronbach α indices of internal consistency for both samples and time points are in Table S1 in Multimedia Appendix 2. The indices for the objective knowledge scale were unacceptable in both samples at T1 (α=.48-.49), and poor to questionable at T2 (α=.54-.62). For the subjective knowledge scale excellent Cronbach α indices of internal consistency were obtained across both samples and time points (α=.92-.95). The DHLI scales reached acceptable to good indices (α=.70-.85), while the VisAWI and SEEQ scales reached good to excellent internal consistency indices (α=.80-.95).

#### Knowledge

**Figure 3:**
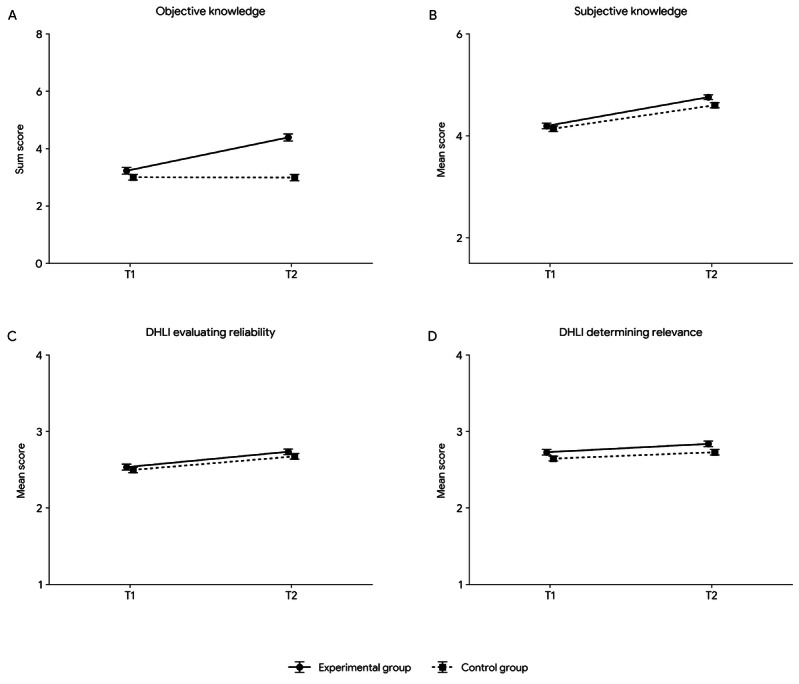
Means and SEs (error bars) in the adolescent sample for the knowledge and digital health literacy scales. DHLI: Digital Health Literacy Instrument.

All descriptive statistics regarding both objective and subjective knowledge are in Table S2 in Multimedia Appendix 2 and Figures 3 (adolescents) and 4 (teachers). Regarding objective knowledge in the adolescents’ sample, significant main effects of both time (*F*_1,498_=67.56, η²_p_=.120; *P*<.001) and group (*F*_1,498_=32.42, η²_p_=.061; *P*<.001), and a significant time×group interaction (*F*_1,498_=70.42, η²_p_=.124; *P*<.001) were observed. Post hoc Bonferroni-corrected pairwise *t* tests revealed a significant knowledge increase from T1 to T2 in the experimental group (*t*_250_=−11.77, *d*=−0.65; *P*<.001) but no significant change in the control group (*t*_248_=0.12, *d*=0.01; *P*=.90). There was a significant group difference at T2 (*t*_498_=8.44, *d*=0.79; *P*<.001), but not at T1 (*t*_498_=1.49, *d*=0.13; *P*=.14).

**Figure 4:**
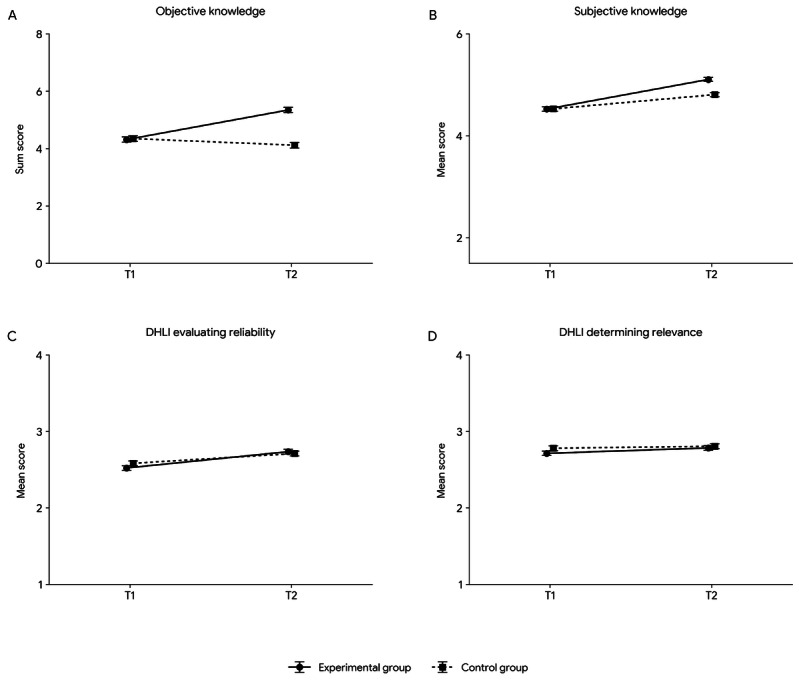
Means and SEs (error bars) in the teacher sample for the knowledge and digital health literacy scales. DHLI: Digital Health Literacy Instrument.

Among teachers, the same pattern of results emerged with significant main effects of time (*F*_1, 601_=52.24, η²_p_=.08; *P*<.001), group (*F*_1,601_=24.10, η²_p_=.039; *P*<.001) and a significant time×group interaction (*F*_1,601_=128.79, η²_p_=.177; *P*<.001). Post hoc Bonferroni-corrected pairwise *t* tests revealed significant increases from T1 to T2 in the experimental group (*t*_300_=−13.12, *d*=−0.63; *P*<.001) as well as a significant decrease in the control group (*t*_301_=2.92, *d*=0.14; *P*=.004). There was a significant group difference at T2 (*t*_601_=9.33, *d*=0.75; *P*<.001) but not at T1 (*t*_601_=−0.24, *d*=−0.02; *P*=.81).

A significant main effect of time emerged regarding subjective knowledge in the adolescents’ sample (*F*_1,498_=207.93, η²_p_=.295; *P*<.001), but there was no significant main effect of group (*F*_1,498_=2.72, η²_p_=.005; *P*=.10), and no time×group interaction (*F*_1,498_=2.04, η²_p_=.004; *P*=.15).

For subjective knowledge among teachers, significant main effects of time (*F*_1,601_=262.11, η²_p_=.304; *P*<.001), group (*F*_1,601_=7.14, η²_p_=.012; *P*=.008), and a significant time×group interaction (*F*_1,601_=32.42, η²_p_=.051; *P*<.001) were observed. Post hoc Bonferroni-corrected pairwise *t* tests revealed a significant increase from T1 to T2 in the experimental group (*t*_300_=−15.46, *d*=−0.78; *P*<.001) as well as the control group (*t*_301_=−7.43, *d*=−0.37; *P*<.001), with a larger effect size in the experimental group. There was a significant group difference at T2 (*t*_601_=5.20, *d*=0.40; *P*<.001) but not at T1 (*t*_601_=−0.08, *d*=−0.01; *P*=.94).

#### Digital Health Literacy

All descriptive statistics regarding the digital health literacy scales are in Table S2 in Multimedia Appendix 2 and Figures 3 (adolescents) and 4 (teachers).

On the DHLI “evaluating reliability” scale a significant main effect of time was observed (*F*_1, 498_=62.92, η²_p_=.112; *P*<.001) in the adolescents’ sample, but there was no significant time×group interaction (*F*_1,498_=0.28, η²_p_<.001; *P*=.60) and no main effect of group (*F*_1, 498_=1.11, η²_p_=.002; *P*=.29). As the interaction effect was not significant, no post hoc *t*-tests were computed.

Among teachers, a significant main effect of time (*F*_1,601_=98.73, η²_p_=.141; *P*<.001) and a significant time×group interaction (*F*_1,601_= 6.21, η²_p_=.010; *P*=.01) were observed. There was no significant main effect of group (*F*_1,601_=0.21, η²_p_<.001; *P*=.65). Post hoc Bonferroni-corrected pairwise *t* tests revealed a significant increase from T1 to T2 in the experimental group (*t*_300_=−8.78, *d*=−0.40; *P*<.001) as well as the control group (*t*_301_=−5.27, *d*=−0.24; *P*<.001), with a larger effect size in the experimental group. There were no significant group differences at T2 (*t*_601_=0.55, *d*=0.05; *P*=.58) nor at T1 (*t*_601_=−1.44, *d*=−0.11; *P*=.15).

On the DHLI “determining relevance” scale significant main effects of time (*F*_1,498_=15.29, η²_p_=.030; *P*<.001) and group (*F*_1,498_= 5.02, η²_p_=.010; *P*=.03) were observed in the adolescents’ sample, but there was no significant time×group interaction (*F*_1,498_=0.25, η²_p_<.001; *P*=.62). As the interaction effect was not significant, no post hoc *t* tests were computed.

In the teachers’ sample, only the main effect of time (*F*_1,601_=8.81, η²_p_=.014; *P*=.003) reached significance for the DHLI “determining relevance” outcome. There was no significant main effect of group (*F*_1,601_=1.26, η²_p_=.002; *P*=.26) and no significant time×group interaction (*F*_1,601_=1.97, η²_p_=.003; *P*=.16). As the interaction effect was not significant, no post hoc *t* tests were computed.

#### Postintervention Evaluation

The descriptive findings regarding the overall quality and visual aesthetics of the short mobile intervention are presented in Table 1. Both samples reported overall quality above the hypothetical mean of the questionnaire. Regarding visual aesthetics, mean values surpassing a threshold of 4.5—indicative of an overall positive evaluation—were obtained in both samples and all subscales.

In terms of intervention characteristics (Table 2), the majority of participants in both samples assessed the length, tempo, and difficulty of the intervention as “exactly right.” Regarding applicability (Table 3), teachers demonstrated high professional acceptance, for example, by reporting willing to recommend the short mobile interventions to their students and colleagues. Willingness to engage with the content in free time or recommendations to private contacts (eg, friends and relatives) was higher among teachers than among adolescents, but was overall only moderate in both samples.

**Table 1:** Descriptive statistics for the quality and user experience scales in the experimental groups in both samples.

User experience scales		Adolescents, mean (SD)	Teachers, mean (SD)
**SEEQ^a^**			
	Learning and value	3.50 (0.91)	3.65 (0.82)
**VisAWI^b^**			
	Overall	4.83 (0.92)	5.13 (0.93)
	Simplicity	4.94 (1.04)	5.28 (1.07)
	Diversity	4.64 (1.08)	4.96 (1.03)
	Colorfulness	4.85 (1.04)	5.04 (0.97)
	Craftsmanship	4.93 (1.02)	5.24 (1.04)

^a^SEEQ: Student Evaluation of Educational Quality.

^b^VisAWI: Visual Aesthetics of Websites Inventory.

**Table 2:** Descriptive statistics regarding length, tempo, and difficulty of the experimental intervention in both samples.

Length, tempo and difficulty scales			Adolescents, n (%)^a^		Teachers, n (%)^a^
**Length**					
	Too short	4 (1.6)		4 (1.3)	
	Exactly right	176 (70.1)		269 (89.4)	
	Too long	71 (28.3)		28 (9.3)	
**Tempo**					
	Too fast	13 (5.2)		6 (2)	
	Exactly right	193 (76.9)		260 (86.4)	
	Too slow	45 (17.9)		35 (11.6)	
**Difficulty**					
	Too easy	44 (17.5)		59 (19.6)	
	Exactly right	172 (68.5)		229 (76.1)	
	Too high	35 (13.9)		13 (4.3)	

^a^Cumulative percentages may exceed or fall below 100% due to rounding.

The descriptive findings regarding implementation intentions (in teachers; Table 3) and motivation (in adolescents; Table 3) support the integration of the intervention into formal education. Specifically, a majority of teachers indicated that they would use the short mobile interventions across different educational contexts, with particularly high endorsement (> 80%) for implementation in classes and during project weeks. Correspondingly, adolescents reported that the use of such tools in various educational contexts would likely be motivating, particularly for homework and in classes (>75%).

**Table 3:** Descriptive statistics regarding applicability, implementation intentions, and motivation for the experimental intervention in both samples.

Applicability, implementation intention and motivation scales			Adolescents, n (%)^a^			Teachers, n (%)^a^	
		No or likely no		Yes or likely yes	No or likely no		Yes or likely yes
**Would you …**							
	… complete mini-learnings^b^ of this kind in your free time?	137 (54.6)		114 (45.4)	127 (42.2)		174 (57.8)
	… recommend mini-learnings of this kind to friends?	138 (55)		113 (45)	142 (47.2)		159 (52.8)
	… recommend mini-learnings of this kind to relatives?	131 (52.2)		120 (47.8)	156 (51.8)		145 (48.2)
	… recommend mini-learnings of this kind to your colleagues?	—^c^		—	65 (21.6)		236 (78.4)
	… recommend mini-learnings of this kind to your students?	—		—	33 (11)		268 (89)
	... use mini-learnings of this kind in class?	—		—	37 (12.3)		264 (87.7)
	... use mini-learnings of this kind in the context of homework?	—		—	81 (26.9)		220 (73.1)
	... use mini-learnings of this kind during project weeks?	—		—	56 (18.6)		245 (81.4)
	... share mini-learnings of this kind in the class chat or digital teaching/learning space?	—		—	73 (24.3)		228 (75.7)
**Would it motivate you if mini-learnings of this kind...**							
	... were used in class?	59 (23.5)		192 (76.5)	—		—
	... were used in the context of homework?	56 (22.3)		195 (77.7)	—		—
	... were used during project weeks?	66 (26.3)		185 (73.7)	—		—
	... were shared in the class chat or digital teaching/learning space?	98 (39)		153 (61)	—		—
	... were recommended to you by your teachers?	86 (34.3)		165 (65.7)	—		—

^a^Cumulative percentages may exceed or fall below 100% due to rounding.

^b^The short mobile intervention was introduced to the participants under the name “mini-learning”.

^c^Not available.

## Discussion

### Overview

This study evaluates an innovative, accessible, and gamified short intervention designed for mobile use (eg, on smartphones and tablets) and flexible deployment in educational settings such as schools. The effectiveness of the intervention was analyzed with a randomized-controlled pre-post approach with an active control group in 2 separate samples, that is, adolescents in secondary school (the main target group of the intervention) and teachers. Consistent with some—but not all—hypotheses, the results showed significant interaction effects (eg, stronger increases in the experimental groups) for objective knowledge, self-reported knowledge, and one facet of digital health literacy in the teacher sample. Among adolescents, a significant interaction effect was observed only for objective knowledge but not for the other three outcomes (including the primary subjective knowledge outcome).

### Key Findings

For the main target group of the intervention, adolescents in secondary school, significantly higher increases could be observed in only one of four outcomes for the experimental group compared to the control group. Specifically, the experimental group’s objective knowledge increased from the baseline to the post-intervention assessment, while there was no such increase in the control group. This finding is consistent with the hypotheses and with prior findings demonstrating positive effects of (more comprehensive) digital health literacy interventions for adolescents in Germany [17-19] and other target groups [41-43]. Overall, our findings concerning objective knowledge underline the potential of using short mobile interventions for the promotion of aspects of digital health literacy. However, contrary to our hypothesis, this objective improvement did not translate into a significant increase in self-reported subjective knowledge among adolescents and was also not observed in the 2 digital health literacy subscales “evaluating reliability” and “determining relevance.” Interestingly, the control group also showed descriptive improvements in subjective knowledge and health literacy following their intervention, which likely explains the lack of statistically significant interactions. There are several potential reasons for this pattern of results: first, the control intervention, a short mobile intervention on a different topic (on average values), might have been too similar to the experimental intervention [44]. Specifically, the control intervention might have also promoted some aspects captured by the knowledge and digital health literacy scales, although it was initially designed to focus on other aspects of digital health literacy, namely numeracy in the context of health information (for example, understanding how the arithmetic mean can be interpreted in health contexts). For example, evaluating health information in graphs might implicitly require understanding averages which was promoted in the control group intervention. To prevent this overlap between groups, future trials should use active control groups with truly distinct intervention topics. Second, expectation, demand, and placebo effects might have inflated the improvements in the control group [45,46]. This is supported by the fact that the lack of statistical interaction effects only occurred in the self-reported outcomes and not the—arguably more objective—knowledge quiz [47]. Third, the scope of the experimental short mobile intervention—due to its intended brevity (10 minutes)—might have been too narrow to promote knowledge and digital health literacy with large effect sizes in this target group. A broad and comprehensive promotion of digital health literacy with mobile interventions might therefore only be achieved when used in blended-learning environments alongside other educational approaches or when multiple modules on similar topics are combined [33].

Evidence of the potential negative effects of smartphone use [48-50], particularly among adolescents, has sparked debate regarding the introduction of school-based smartphone bans [51]. However, restrictive device bans do not resolve these issues entirely, as mobile technology has become deeply integrated into everyday life. Consequently, educating adolescents on the responsible use of these devices is a vital strategy for enhancing both digital literacy and digital health literacy [5,52,53]. Importantly, the flexibility of the intervention evaluated in this study makes it applicable even in schools with comprehensive smartphone bans, for example, by being presented on school-supplied hardware, such as tablets or laptops.

In contrast to the adolescents, in the teachers’ sample, the effects were more robust. Specifically, statistically significant interaction effects were observed for both objective and subjective knowledge and the “evaluating reliability” subscale of digital health literacy (though not fully supported by the post hoc tests for the latter). The only outcome without any significant interaction effects was the “determining relevance” subscale of the DHLI. As for this subscale, no significant results could be obtained in either sample, it is possible that it did not adequately measure the specific dimensions of digital health literacy targeted by the intervention. Future development of intervention modules should more explicitly address the gap between the information presented and the user’s perceived personal applicability, as this is an integral aspect of digital health literacy [6,7]. The observation that subjective knowledge effects were more robust in the teachers’ sample than among adolescents might be explained by teachers estimating their baseline level of knowledge more realistically, whereas adolescents may have overestimated theirs [54,55]. This interpretation is corroborated by the convergence of qualitative findings for objective and subjective knowledge among teachers, a pattern not observed in the adolescent sample. Further evidence for this interpretation comes from the baseline data: whereas adolescents showed descriptively lower levels of objective knowledge than teachers, the difference between the samples’ subjective knowledge was notably smaller. Teachers might have had relevant prior knowledge and abstract reasoning skills [56] on which they could build to efficiently learn the contents of the mobile intervention. Another possible reason for the discrepancies between the adolescents’ and the teachers’ findings is that teachers were more motivated given their professional interest in the intervention. This is partly supported by the fact that the teachers reported higher levels of motivation and implementation intentions in the postintervention survey than adolescents.

Overall, the largely positive evaluation results in the teachers’ sample provide important evidence for the potential of the short mobile intervention to be used in an educational context. However, these results also indicate that the intervention works better for older and more educated individuals than for the main target group. This highlights that future revisions and newly developed similar interventions should aim for a tighter alignment with the primary target group’s baseline abilities.

Across both samples, the intervention was met with high overall acceptance and positive aesthetic evaluations, underscoring the value of high-quality design in digital learning tools [39]. In addition, the majority of the teacher and adolescent participants found the tempo, length, and difficulty of the intervention appropriate. While endorsement of the intervention for private use was only moderate in both samples, teachers reported high willingness to use the intervention in professional—that is, educational—contexts, for example, by recommending them to colleagues and students, and by implementing them in classes and project weeks. Furthermore, the majority of adolescents confirmed that such integration would enhance their motivation. Taken together, these descriptive findings highlight the aesthetic and educational quality of the intervention, supporting its potential for effective integration into secondary educational settings. The gamification elements incorporated in the intervention may have enhanced the motivation of the adolescents and strengthened the teachers’ implementation intentions [29,30,57]. Moreover, the intervention’s brevity, coupled with its alignment with German media, information, and digital literacy curricula, further underscores its broad applicability and feasibility within educational settings [20]. Hence, the results of this evaluation highlight that the short mobile interventions could be used to promote aspects of digital health literacy as required by national legislation in Germany, such as the German Social Code Book V [15,16].

### Limitations

A few limitations should be noted when interpreting the results of the study. First, as outlined above, the control group received an intervention highly similar to the experimental group intervention, introducing a risk of confounding. Second, because of the brevity of the intervention (which was intended to secure its flexible use in diverse school contexts), only a few aspects of digital health literacy were focused on here, namely data—and more specifically—graph literacy. While these are integral and often overlooked elements of digital health literacy education [23], the comprehensive promotion of (digital) health literacy in schools should more broadly cover all of its central elements [12]. This aim might be achieved by combining multiple e-learning modules, such as the examples presented in this research, with other didactic approaches, for example, educational videos [58,59] and classic classroom teaching materials such as worksheets and discussions. Third, the outcomes of the present evaluation partly relied on self-reports, which may have introduced biases due to impression management and limited introspection abilities [60]. Fourth, the reliability of the objective knowledge quiz was very low, possibly due to the difficulty of some items, the diversity of the content captured by a single scale, and the relatively strict scoring scheme. In the future, more thorough pretesting with individuals from the target group might help to improve the scale’s quality. Fifth, the short retest interval precludes the interpretation of long-term changes in digital health literacy and thus does not allow for testing the stability of the effects, which should be addressed in more comprehensive future studies. Sixth, the intervention was tested online in a setting that offered less control than in a laboratory-based experiment. Some aspects of school environments, such as distractions or technical problems, might not be well represented in this setting. In the future, the short mobile intervention should therefore also be tested in environments with higher ecological validity, such as classrooms. Finally, the DHLI instrument was adapted to match the contents of the evaluated intervention. Results are therefore not comparable to other studies using the same instrument.

### Conclusions

Overall, the results highlight the potential of using short mobile interventions in schools to promote aspects of digital health literacy, for example, regarding knowledge on the interpretation of health-related graphs, thereby contributing to public health endeavors to promote digital health literacy. However, as not all relevant outcomes (including the primary outcome) showed statistically significant results, the intervention should be further optimized and refined in the future to secure more comprehensive knowledge and competence gains in adolescents. Schools should consider using short, gamified mobile interventions like the one tested here as feasible, low-cost tools to teach specific digital health literacy skills. However, to change students’ broader health literacy competencies and knowledge, these modules are likely best used as part of a sequenced curriculum, not as stand-alone solutions.

## Appendix

Multimedia Appendix 1 Original items used to assess subjective and objective knowledge.

Multimedia Appendix 2 Additional tables for the randomized controlled trial of a short mobile intervention on digital health literacy in adolescents and teachers.

## Abbreviations

DHLI: Digital Health Literacy Instrument
SEEQ: Student Evaluation of Educational Quality
VisAWI: Visual Aesthetics of Websites Inventory

## Data Availability

The datasets generated and analyzed during this study are available from the *Stiftung Gesundheitswissen* on reasonable request.
