# Supplementary material for: Effects of a Short Mobile Intervention on Digital Health Literacy in Adolescents and Teachers: Randomized Controlled Trial
Source: J Med Internet Res. 2026 Jul 21;28:e96284. doi: 10.2196/96284 (PMC13439045; doi:10.2196/96284)
Supplement: Supplementary file 1 [file jmir_v28i1e96284_app1.pdf]

Effects of a Short Mobile Intervention on Digital Health Literacy in Adolescents and Teachers: Randomized Controlled Trial

Supplementary Material 1: Original items used to assess subjective and objective knowledge

Subjective knowledge

**General instruction** (original German *[English translation])*
Inwieweit stimmen Sie *(stimmst du)* den folgenden Aussagen zu?<br>Bitte beantworten Sie alle Fragen, auch wenn Sie sich unsicher sind. Wählen Sie in diesem Fall die am ehesten auf Sie zutreffende Antwort.<br><br>*[To what extent do you agree with the following statements?<br>Please answer all questions even if you are unsure. In that case, select the answer that best applies to you.]*

Items

**No.**	**Item code**	**Item** (original German *[English translation])*
1	SW01	Ich kann erklären, welche Elemente eine gute Grafik ausmachen.<br>*[I can explain the elements that make up a good graph.]*
2	SW02	Ich kenne die Kriterien, mit deren Hilfe man eine Grafik bezüglich ihrer Qualität bewerten kann.<br>*[I know the criteria used to evaluate a graph‘s quality.]*
3	SW03	Ich kann eine gute Grafik von einer weniger guten Grafik unterscheiden.<br>*[I can differentiate a good graph from a poorer graph.]*
4	SW04	Ich kann erklären, wie das Verändern von bestimmten Elementen in einer Grafik ihre Interpretation beeinflusst.<br>*[I can explain how changing the elements in a graph influences its interpretation .]*
5	SW05	Ich kann erklären, warum das Erkennen von guten Grafiken wichtig für meine Gesundheit sein kann.<br>*[I can explain why recognizing good graphs may be important for my health.]*
6	SW06	Ich kann erkennen, ob die Daten in Grafiken dramatisiert oder neutral dargestellt sind.<br>*[I can recognize whether the data in a graph is presented in a dramatized or neutral way.]*
7	SW07	Ich kann erklären, was eine gute Überschrift einer Grafik ausmacht.<br>*[I can explain what constitutes a good title for a graph.]*
8	SW08	Ich kann erklären, was eine gute Achseneinteilung einer Grafik ausmacht.<br>*[I can explain what constitutes a good axis scale on a graph.]*
9	SW09	Ich kenne den Nutzen von Achsenbeschriftungen bei Grafiken.<br>*[I know the benefits of labeling axis on graphs.]*

10	SW10	Ich kenne die Funktion von Quellenangaben bei Grafiken.<br>*[I know the purpose of reference information in graphs.]*
11	SW11	Ich kann erklären, was eine angemessene Farbgebung bei Grafiken ausmacht.<br>*[I can explain what constitutes appropriate coloring in graphs.]*
12	SW12	Ich kann erklären, wie unterschiedliche Perspektiven (z. B. 3D-Effekte) Grafiken beeinflussen.<br>*[I can explain how different perspectives (e.g., 3D effects) influence graphs.]*

Response options

**Response codes**	**Response option** (original German *[English translation]*)
1	stimme überhaupt nicht zu<br>*[strongly disagree]*
2	stimme nicht zu<br>*[disagree]*
3	stimme eher nicht zu<br>*[somewhat disagree]*
4	stimme eher zu<br>*[somewhat agree]*
5	stimme zu<br>*[agree]*
6	stimme voll und ganz zu<br>*[strongly agree]*

Objective knowledge quiz

**General instruction** (original German *[English translation]*)
Bitte markieren Sie *(markiere)* bei den folgenden Fragen alle zutreffenden Antworten. Bei jeder Frage trifft mindestens eine Antwort zu. Es können allerdings auch zwei, drei oder alle vier Antworten zutreffen.<br><br>*[For the following questions, please mark all correct responses. In each question, at least one response option is correct. It is also possible that two, three or all four response options are correct.]*

Question KW01

**Instruction**
Oftmals werden Informationen rund um das Thema Gesundheit grafisch dargestellt, z. B. mithilfe von Diagrammen oder Abbildungen.<br>Was macht eine gute Grafik zu Gesundheitsthemen aus?<br>*[Information about health are often presented in graphs, e.g., in diagrams or illustrations. What constitutes a good graph on health-related topics?]*

**No.**	**Response option code**	**Response option**	**Correct response**
1	KW01A	Dass sie möglichst bunt und kreativ gestaltet ist.<br>*[That it is designed to be as colorful and creative as possible.]*	
2	KW01B	Dass sie eine spannende Überschrift hat.<br>*[That it has an exciting title.]*	
3	KW01C	Dass eine Quelle angegeben ist.<br>*[That it has reference information.]*	x
4	KW01D	Dass sie verschiedene visuelle Effekte nutzt.<br>*[That it uses different visual effects.]*	

Question KW02

**Instruction**
Welches dieser Kriterien ist KEIN Kriterium bzw. welche dieser Kriterien sind KEINE Kriterien für eine gute Grafik zu Gesundheitsthemen?<br>*[Which of the following criteria is not a criterion or which are not criteria for a good graph on health-related topics?]*

**No.**	**Response option code**	**Response option**	**Correct response**
1	KW02A	Unregelmäßige Achseneinteilung<br>*[Irregular axis scales]*	x
2	KW02B	3D-Effekte<br>*[3D effects]*	x
3	KW02C	Neutrale Überschrift<br>*[Neutral title]*	
4	KW02D	Quellenangaben<br>*[Reference information]*	

Question KW03

Instruction
Welche Aussage zu Quellenangaben trifft zu bzw. welche Aussagen zu Quellenangaben treffen zu?<br>*[Which statement about references is correct or which statements about references are correct?]*

No.	Response option code	Response option	Correct response
1	KW03A	Auch ohne Quellenangaben lässt sich nachvollziehen, woher die Daten kommen und wie sie ermittelt wurden.<br>*[It is easy to retrace where data comes from and how it was obtained even without reference information.]*	
2	KW03B	Quellenangaben geben den Ursprung der Daten an und helfen, das Dargestellte bewerten zu können.<br>*[Reference information gives details on the origin of the data and helps to evaluate what is depicted.]*	x
3	KW03C	Auf Quellenangaben bei Grafiken kann verzichtet werden, solange die Daten korrekt dargestellt sind.<br>*[Reference information on graphs can be omitted as long as the data is depicted correctly.]*	
4	KW03D	Quellenangaben helfen, die Qualität der in Grafiken dargestellten Informationen besser einzuschätzen.<br>*[Reference information on graphs helps to better assess the quality of the information presented in the graph.]*	x

Question KW04

**Instruction**
Welche Aussage zu Überschriften von guten Gesundheitsgrafiken trifft zu bzw. welche Aussagen zu Überschriften von guten Gesundheitsgrafiken treffen zu? *[Which statements about titles of good health graphs is correct or which statements about titles of good health graphs are correct?]*

**No.**	**Response option code**	**Response option**	**Correct response**
1	KW04A	Die Überschrift einer guten Gesundheitsgrafik sollte möglichst dramatisch wirken, damit viele Menschen sich die Grafik anschauen. *[The title of a good health graph should appear as dramatic as possible so that many people look at the graph.]*	
2	KW04B	Eine wertende Überschrift hilft mir, das dargestellte Problem sofort zu erkennen. *[An opinionated title helps me to immediately recognize the problem depicted.]*	
3	KW04C	Es spielt keine Rolle, wenn die Überschrift einer Grafik übertrieben ist, da die Grafik alle Fakten darstellt. *[It doesn't matter if the title of a graph is exaggerated as the graph presents all the facts.]*	
4	KW04D	Eine Überschrift sollte den Inhalt einer Grafik neutral beschreiben, selbst wenn sie langweilig ist. *[A title should describe the contents of a graph in a neutral way even if it sounds boring.]*	x

Question KW05

Instruction
Welche Aussage trifft zu bzw. welche Aussagen treffen zu?<br>*[Which statement is correct or which statements are correct?]*

No.	Response option code	Response option	Correct response
1	KW05A	Ein entscheidendes Kriterium für eine gute Grafik ist, dass die Farben sich nicht beißen.<br>*[A crucial criterion for a good graph is that the colors do not clash.]*	
2	KW05B	3D-Effekte können dabei helfen, die in einer Grafik dargestellten Ergebnisse klarer darzustellen.<br>*[3D effects can help to present the results in a graph in a more precise way.]*	
3	KW05C	In Grafiken verwendete Farben sollten gut unterscheidbar sein.<br>*[The colors used in graph should be easily distinguishable.]*	x
4	KW05D	Die Farben und Perspektiven können beim Erstellen von Grafiken frei gewählt werden, weil sie die Interpretation der dargestellten Daten nicht beeinflussen.<br>*[The colors and perspectives used in a graph can be freely chosen because they do not influence the interpretation of the data depicted.]*	

Question KW06

**Instruction**
Welche Aussage zu Achsen in einer Grafik trifft zu bzw. welche Aussagen zu Achsen in einer Grafik treffen zu?<br>*[Which statement about axis on a graph is correct or which statements about axis on a graph are correct?]*

**No.**	**Response option code**	**Response option**	**Correct response**
1	KW06A	Die Abstände zwischen den Achsenabschnitten sollten möglichst klein sein.<br>*[The intervals between axis ticks should be as small as possible.]*	
2	KW06B	Die Abstände zwischen den Achsenabschnitten sollten möglichst gleich groß sein.<br>*[The intervals between axis ticks should preferably be equal.]*	x
3	KW06C	Auf eine Achsenbeschriftung kann verzichtet werden, wenn klar ist, welche Einheiten gemeint sind.<br>*[Labeling axis can be omitted as long as the intended units are clear.]*	
4	KW06D	Die Interpretation der Ergebnisse kann unterschiedlich ausfallen, je nachdem, wo die Achse beginnt.<br>*[The interpretation of the results can differ depending on where the axis begins.]*	x

Question KW07

**Instruction**
Welcher Aussage zu der folgenden Grafik trifft zu bzw. welche Aussagen zu der folgenden Grafik treffen zu?<br>*[Which statement about the following graph is correct or which statements about the following graph are correct?]*

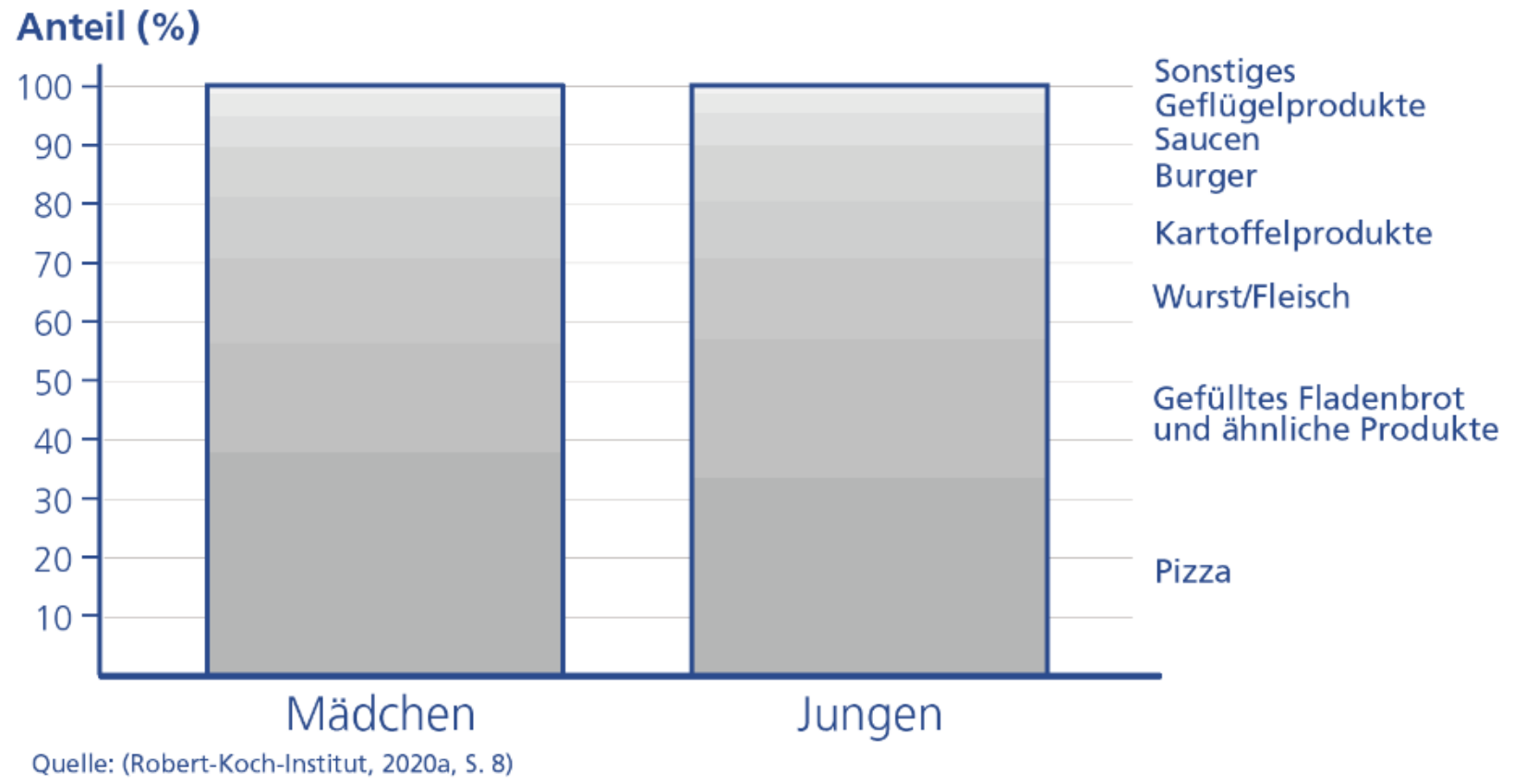

No.	Response option code	Response option	Correct response
1	KW07A	Die Darstellung der verschiedenen Kategorien ist angemessen, da die Einteilung rechts erklärt ist.<br>*[The presention of the different categories is appropriate as the grouping is explained on the right.]*	
2	KW07B	Die Farbabstufungen sollten deutlicher zu unterscheiden sein, um die Grafik angemessen interpretieren zu können.<br>*[The different color shades should be more clearly distinguishable in order to interpret the graph appropriately.]*	x
3	KW07C	Dass die Abbildung keine Überschrift hat, ist kein Problem, weil man auch ohne diese gut erkennt, was dargestellt ist.<br>*[The fact that the graph does not have a title is not a problem as it is easy to recognize what is depicted.]*	
4	KW07D	Für die Qualität der Grafik spricht, dass sie eine seriöse Quellenangabe hat.<br>*[The fact that the graph comes with reputable reference information speaks for its quality.]*	x

Question KW08

Instruction
Welche Aussage zu der folgenden Grafik trifft zu bzw. welche Aussagen zu der folgenden Grafik treffen zu?<br>*[Which statement about the following graph is correct or which statements about the following graph are correct?]*

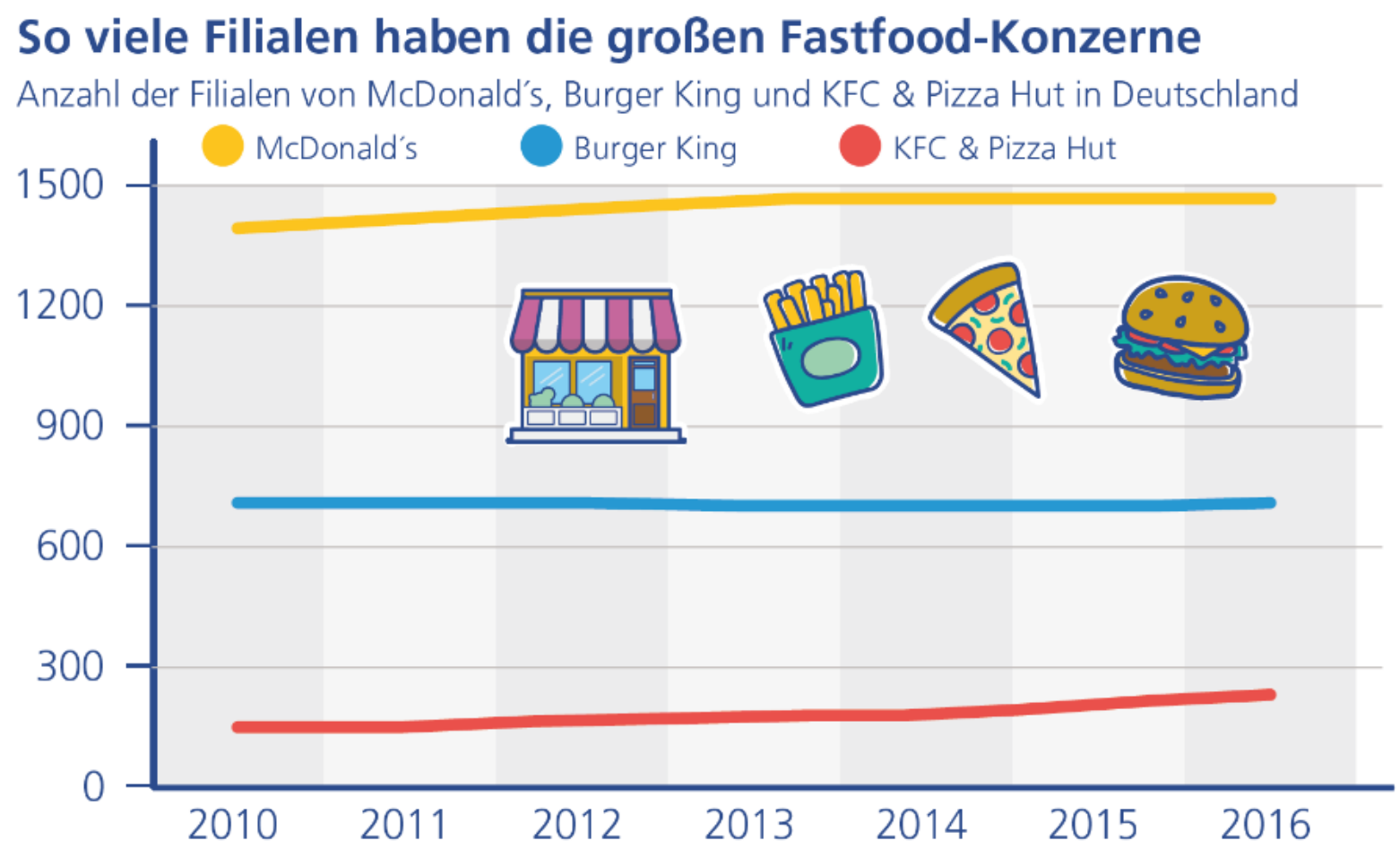

No.	Response option code	Response option	Correct response
1	KW08A	Dass bei dieser Grafik keine Achsenbeschriftungen vorliegen, ist unproblematisch, weil sich aus den anderen Informationen ergibt, was dargestellt ist.<br>*[The fact that the graph does not not have axis labels is not a problem because it is clear what is depicted based on the other information available.]*	
2	KW08B	Bei Grafiken zu großen Unternehmen kann wie hier auf eine Quellenangabe verzichtet werden, weil die Unternehmen ihre Daten sowieso öffentlich zugänglich machen müssen.<br>*[Reference information can be omitted in graphs about large companies (as presented here) because these companies are required to make their data publicly available anyway.]*	
3	KW08C	Dass die x-Achse nicht bei Null beginnt, ist hier kein Problem, weil die Ergebnisse dadurch nicht dramatisiert werden.<br>*[The fact that the x-axis does not start at zero is not a problem because the results are not dramatized.]*	x
4	KW08D	Auch wenn die Farben in der Grafik nicht den Farben der Logos der Unternehmen entsprechen, ist die Darstellung angemessen.<br>*[The presentation is appropriate even if the colors in the graph do not match the colors of the companies' logo.]*	x
