# Supplementary material for: Effects of a Short Mobile Intervention on Digital Health Literacy in Adolescents and Teachers: Randomized Controlled Trial
Source: J Med Internet Res. 2026 Jul 21;28:e96284. doi: 10.2196/96284 (PMC13439045; doi:10.2196/96284)
Supplement: Supplementary file 2 [file jmir_v28i1e96284_app2.pdf]

Effects of a Short Mobile Intervention on Digital Health Literacy in Adolescents and Teachers: Randomized Controlled Trial

Supplementary Material 2: Supplementary Tables

Supplementary Table 1: Cronbach's α indices of internal consistency across the questionnaire scales and samples.

Questionnaire scale	Number of items	Sample	Time point	Cronbach's α
**Objective knowledge quiz**	8	Adolescents	T1	.48
			T2	.62
		Teachers	T1	.49
			T2	.54
**Subjective knowledge questionnaire**	12	Adolescents	T1	.92
			T2	.95
		Teachers	T1	.93
			T2	.95
**DHLI reliability**	3	Adolescents	T1	.74
			T2	.81
		Teachers	T1	.72
			T2	.79
**DHLI relevance**	3	Adolescents	T1	.70
			T2	.81
		Teachers	T1	.76
			T2	.85
**SEEQ**	5	Adolescents	T2	.83
		Teachers	T2	.81
**VisAWI total score**	18	Adolescents	T2	.94
		Teachers	T2	.95
**VisAWI simplicity**	5	Adolescents	T2	.85
		Teachers	T2	.90
**VisAWI diversity**	5	Adolescents	T2	.85
		Teachers	T2	.89
**VisAWI colorfulness**	4	Adolescents	T2	.84
		Teachers	T2	.84
**VisAWI craftmanship**	4	Adolescents	T2	.80
		Teachers	T2	.84

Supplementary Table 2: Means (M) and standard deviations (SD) for the questionnaire scales.

**Adolescents**	**Experimental group**		**Control group**	
	**T1** ***M (SD)***	**T2** ***M (SD)***	**T1** ***M (SD)***	**T2** ***M (SD)***
Objective knowledge	3.22 (1.80)	4.39 (1.94)	3.00 (1.59)	2.98 (1.77)
Subjective knowledge	4.19 (0.86)	4.76 (0.75)	4.14 (0.87)	4.60 (0.81)
DHLI evaluating reliability	2.53 (0.62)	2.73 (0.58)	2.50 (0.55)	2.67 (0.57)
DHLI determining relevance	2.73 (0.58)	2.84 (0.57)	2.64 (0.50)	2.73 (0.56)
**Teachers**	**Experimental group**		**Control group**	
	T1 ***M (SD)***	***T2*** ***M (SD)***	***T1*** ***M (SD)***	**T2** ***M (SD)***
Objective knowledge	4.31 (1.59)	5.34 (1.51)	4.34 (1.75)	4.12 (1.71)
Subjective knowledge	4.52 (0.78)	5.10 (0.68)	4.53 (0.80)	4.81 (0.73)
DHLI evaluating reliability	2.52 (0.51)	2.74 (0.53)	2.58 (0.53)	2.71 (0.56)
DHLI determining relevance	2.71 (0.49)	2.78 (0.52)	2.78 (0.54)	2.81 (0.55)

Note: objective knowledge scale range: 0–8; subjective knowledge scale range: 1–6; Digital Health Literacy Instrument (DHLI) scale range: 1–4.
